# Thermofluor-Based Optimization Strategy for the Stabilization of Recombinant Human Soluble Catechol-*O*-Methyltransferase

**DOI:** 10.3390/ijms232012298

**Published:** 2022-10-14

**Authors:** Ana M. Gonçalves, Augusto Q. Pedro, Diana M. Oliveira, Adriana E. Oliveira, Marino F. A. Santos, Márcia A. S. Correia, João A. Queiroz, Eugénia Gallardo, Maria J. Romão, Luís A. Passarinha

**Affiliations:** 1 CICS-UBI—Health Sciences Research Centre, University of Beira Interior, 6201-506 Covilhã, Portugal; 2 Associate Laboratory i4HB-Institute for Health and Bioeconomy, NOVA School of Science and Technology, Universidade NOVA de Lisboa, 2819-516 Caparica, Portugal; 3 UCIBIO-Applied Molecular Biosciences Unit, Chemistry Department, NOVA School of Science and Technology, Universidade NOVA de Lisboa, 2829-516 Caparica, Portugal; 4 Chemistry Department, CICECO-Aveiro Institute of Materials, University of Aveiro, Campus Universitário de Santiago, 3810-193 Aveiro, Portugal; 5 Laboratório de Fármaco-Toxicologia, UBI Medical, Universidade da Beira Interior, 6201-506 Covilhã, Portugal

**Keywords:** protein activity, protein stability, soluble catechol-*O*-methyltransferase (SCOMT), thermal shift assay (TSA)

## Abstract

Catechol-*O*-methyltransferase (COMT) has been involved in a number of medical conditions including catechol-estrogen-induced cancers and a great range of cardiovascular and neurodegenerative diseases such as Parkinson’s disease. Currently, Parkinson’s disease treatment relies on a triple prophylaxis, involving dopamine replacement by levodopa, the use of aromatic L-amino acid decarboxylase inhibitors, and the use of COMT inhibitors. Typically, COMT is highly thermolabile, and its soluble isoform (SCOMT) loses biological activity within a short time span preventing further structural and functional trials. Herein, we characterized the thermal stability profile of lysate cells from *Komagataella pastoris* containing human recombinant SCOMT (hSCOMT) and enzyme-purified fractions (by Immobilized Metal Affinity Chromatography—IMAC) upon interaction with several buffers and additives by Thermal Shift Assay (TSA) and a biological activity assessment. Based on the obtained results, potential conditions able to increase the thermal stability of hSCOMT have been found through the analysis of melting temperature (T_m_) variations. Moreover, the use of the ionic liquid 1-butyl-3-methylimidazolium chloride [C_4_mim]Cl (along with cysteine, trehalose, and glycerol) ensures complete protein solubilization as well as an increment in the protein Tm of approximately 10 °C. Thus, the developed formulation enhances hSCOMT stability with an increment in the percentage of activity recovery of 200% and 70% when the protein was stored at 4 °C and −80 °C, respectively, for 12 h. The formation of metanephrine over time confirmed that the enzyme showed twice the productivity in the presence of the additive. These outstanding achievements might pave the way for the development of future hSCOMT structural and biophysical studies, which are fundamental for the design of novel therapeutic molecules.

## 1. Introduction

Laboratory techniques to determine enzymatic activity, protein–ligand interactions, and protein 3D structure, require stable samples over long periods of time [1]. However, it is known that a significant number of enzymes, including catechol-*O*-methyltransferase (COMT) isoforms, lose their activity in a short period of time even at physiological temperatures [2]. Responsible for the *O*-methylation of molecules with catechol moieties, such as the neurotransmitter dopamine, COMT has been linked to several neurodegenerative disorders, with Parkinson’s disease being one of the most studied. [3]. Nowadays, the inhibition of COMT is seen as a viable option to treat Parkinson’s disease and, in combination with levodopa, different inhibitors have been proposed for this purpose. Therefore, finding new safe and potent COMT inhibitors is essential. The characterization of putative drug candidates requires high amounts of purified and stable COMT isoforms to be used in proper analytical and biophysical techniques.

Several factors could affect protein stability including buffers, salts, detergents, and ligands [4]. Typically, protein denaturation is prevented by the addition of stabilizers (e.g., sugars, reducing agents, and cryoprotectants), avoiding aggregation and promoting stability [1,5].

The use of sugars (e.g., sucrose, glucose, and trehalose) is reported to influence the protein–solvent interaction with a direct effect on both tertiary and quaternary protein structures [6,7,8]. Specifically, Lerbret and coworkers observed the “destructuring” effect of trehalose upon the water network by which the water molecules are ordered next to the sugar molecule (as a kosmotrope), preventing ice formation [9]. Glycerol is also known for its cryoprotectant role [10,11]. This polyol enhances protein stability by increasing its hydration. [12,13]. Finally, it is also reported that the addition of reducing agents (e.g., DTT) to a protein sample prevents the formation of non-native disulfide bonds hampering the formation of putative protein aggregates [14].

Protein stability relies on the respective unfolding Gibbs free energy, ∆G, a temperature-dependent parameter [15]. Most commonly, protein stability decreases with the increase in temperature. As temperature increases, ΔG becomes zero at a so-called melting temperature (T_m_) at which similarly folded and unfolded protein concentrations are present [15,16,17,18]. Compound binding to proteins often leads to a ∆G increase and the concomitant improvement of T_m_ [19].

The fluorescence-based Thermal Stability Assay (TSA) or Differential Scanning Fluorimetry (DSF) was developed by Pantoliano and coworkers and later became known by the term thermofluor [20]. Briefly, TSA relies on the use of a fluorescent dye: the protein hydrophobic core is exposed by temperature-induced denaturation, and the dye will bind to the exposed hydrophobic regions and become fluorescent [21,22] Therefore, when the protein is unfolded, the fluorescence signal significantly increases over a short temperature range and the obtained sigmoidal curve allows the T_m_ value to be calculated, which can also be derived by calculating the peak of the respective first derivate [21,23]. A positive shift in T_m_ can be coupled to an increase in the structural order and a reduced conformational flexibility, while a negative T_m_ variation suggests protein structural changes towards a more disordered conformation [3]. TSA was originally developed for drug-discovery applications as it rapidly screens molecules able to stabilize the protein of interest from large compound libraries. Currently, TSA is routinely used to assess protein stability by systematically testing a great range of buffers and additives (e.g., salts, amino acids, sugars, polyols, reducing agents, and ionic liquids). Therefore, TSA is a valuable tool for different purposes, including the optimization of purification protocols, the detection of protein–ligand interactions, and the optimization of crystallization conditions [24]. However, despite the versatility of TSA, there is no exhaustive study to enlighten the ideal buffer conditions for SCOMT to ensure protein stability.

Herein, taking advantage of the TSA methodology, we aim to screen the soluble isoform of COMT against generic buffer formulations to identify stabilizing conditions, comprising salts, the ideal pH, and simple additives (namely, cysteine, trehalose, glycerol, and ionic liquids), which have already successfully been explored with the membrane COMT isoform [25]. Considering the similarities shared by both isoforms, Mg^2+^ and S-Adenosyl-L-methionine (SAM) dependency, as well as an identical catalytic mechanism, SCOMT (24.7 kDa, 221 residues) has higher K_m_ and V_max_ values, being responsible for the *O*-methylation of catechols at a higher concentration in the peripheric tissues [26]. Starting from the premise that the membrane isoform of COMT present in the lysates of *Komagataella pastoris* can be stabilized by ionic liquids, reducing agents, and sugars, we intend now to adapt and design a new stabilization buffer for the soluble isoform of COMT, evaluating its thermostability, yield, and purity.

## 2. Results and Discussion

COMT is a highly thermolabile enzyme with evidence pointing to a loss of its biological function in a range of 50 to 70% in less than 24 h at 4 °C [2,27].

### 2.1. Stabilizer Effects on Recombinantly Biosynthesized hSCOMT

Previous stabilization studies carried out for hSCOMT lysates obtained from *Escherichia coli* showed that DTT and glycerol were effective to stabilize the protein, maintaining its activity [28]. Moreover, a low concentration of [Ch][DHP] 7.5 mM incorporated in the defined multicompetent buffer (150 mM NaCl, 1 mM MgCl_2_, and 50 mM Tris-Cl, pH 8.0) for a period of 32.4 h at −80 °C has been shown as the ideal conditions to maximize the activity recovery of hMBCOMT [25]. In fact, all the results obtained for recombinant human COMT lysates were helpful to define the proper stabilizers and respective concentrations that should be used in the development of a further formulation to maintain and/or improve hSCOMT stability after the main purification step, as described by our research group [29,30].

Typically, the protein stability was measured by the respective enzyme activity using an HPLC instrument coupled to an electrochemical or coulometric detector as mentioned in the Materials and Methods section. Therefore, the hSCOMT activity in *Komagataella pastoris (K. pastoris)* lysates was assessed after incubation at 4 °C for 12 h with different target stabilizer concentrations.

**Figure 1:**
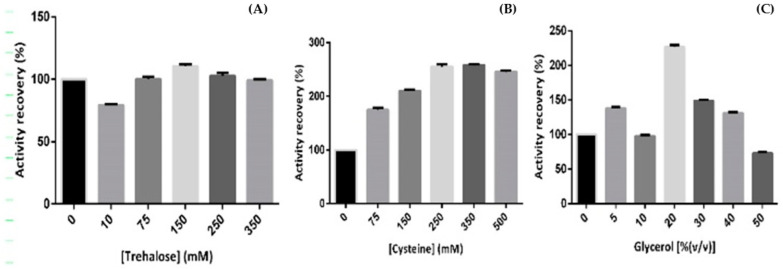
Percentage of hSCOMT activity recovery in the presence and absence of (**A**) trehalose (0 to 350 mM); (**B**) cysteine (0 to 500 mM); (**C**) glycerol (0 to 50%) during a 12 h period. Each value represents the mean of 3 independent samples.

Trehalose was firstly evaluated due to its already-mentioned capability to manipulate water and abrogate protein aggregation by interfering with intermolecular disulfide bond formation [7,31]. The obtained results suggest that 75 to 250 mM trehalose has a higher percentage of hSCOMT activity recoveries (Figure 1A).

**Figure 2:**
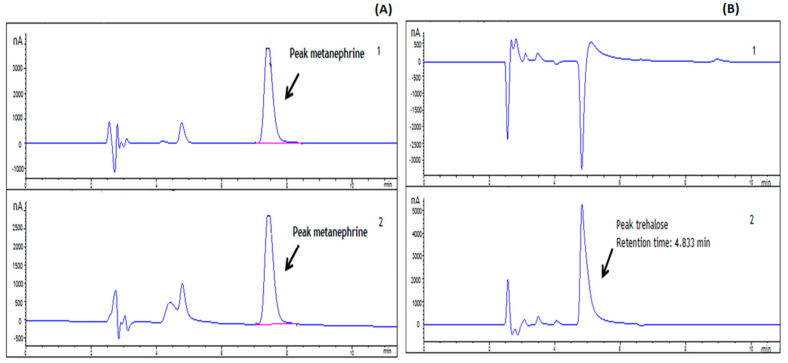
(**A**) Chromatogram of a metanephrine standard; (**B**) Chromatogram of a 300 mM trehalose standard. (**1**) Oxidation potential (+410 mV) and (**2**) reduction potential (−350 mV).

The next step was to evaluate if trehalose could interfere with the HPLC-reversed phase chromatographic retention time of metanephrine (MN), a product correlated with hSCOMT methylating efficiency and used as a viable method to evaluate hSCOMT-specific activity [32]. Typically, the retention time of MN is around 8 min and our results showed that the main peak of trehalose does not show any overlap with MN (Figure 2).

The reducing agent DTT, often used to prevent protein aggregates by reducing disulfide bonds and promoting the correct conformation of hSCOMT, was also tested. [27]. It should be highlighted that the effects of DTT are debated: while some authors defend that concentrations up to 4 mM DTT prevent protein aggregation [33,34], some others defend that DTT can compromise protein activity by binding to the active site [35]. Similar to DTT, cysteine plays a relevant role in the formation of disulfide bonds controlling the correct conformation and the concomitant enzyme activity [36].

**Figure 3:**
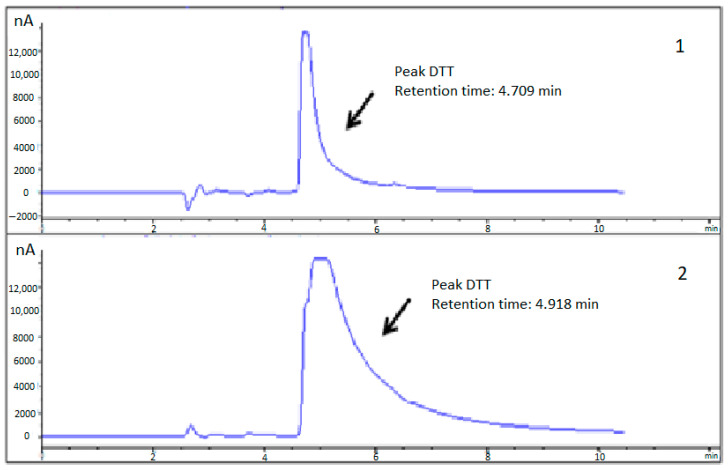
Chromatogram of a 100 mM DTT standard: (**1**) Oxidation potential (+410 mV) and (**2**) Reduction potential (−350 mV).

The specific activity and recovery of COMT activity in the presence and absence of DTT and cysteine (12 h) was now analyzed: DTT (ranging from 50 to 100 mM) has shown to be more effective to maintain hSCOMT activity than cysteine that requires higher concentrations (between 200 and 350 mM) to achieve similar results (Figure 1B). Nevertheless, the coulometric peak exhibited by 100 mM DTT masks the MN peak due to peak drag (Figure 3). Therefore, we decided to exclude the use of DTT in further stabilization studies investing in the use of cysteine that has a similar action, not compromising an MN accurate quantification in more complex samples.

It is important to note that, despite the interference with the MN assessment, the presence of lower concentrations of DTT is important in the initial phase of the bioprocess, namely, in the lysis buffer: complete DTT removal led to a severe decrease in the initial hSCOMT biological activity. As a partially denatured and inactive hSCOMT sample hardly can be restored into a highly biological active state, we decided to preserve the DTT in the initial lysis buffer, reducing its concentration to 10 mM. In fact, at these lower concentrations, DTT was shown to not interfere with the MN peak (data not shown).

Finally, the effect of glycerol on the specific activity of hSCOMT was also analyzed and the activity recovery was assessed (Figure 1C). As previously mentioned, glycerol is often used as a cryoprotectant, increasing the melting temperature, avoiding protein thermal inactivation, and contributing to keep the properties of the native biomolecule [36,37]. Here, the obtained results demonstrate that 20% (*v*/*v*) glycerol allowed the highest activity recovery of hSCOMT. Moreover, no interferences were detected when a cysteine solution (300 mM) or a glycerol solution (5% (*v*/*v*)) was directly injected into the HPLC system.

Based on these initial results, the following concentration ranges were defined—75–250 mm trehalose, 100–150 mm cysteine, and 10–30% (*v*/*v*) glycerol—as the starting point for the stabilization of hscomtval108-6his. These concentrations were applied to cell lysates and to fractions containing the target enzyme from immobilized-metal affinity chromatography (IMAC). IMAC was revealed to be extremely efficient and selective, allowing the recovery (using 300 mM of imidazole) of a pure hSCOMT sample (purification fold: 81; bioactivity recovery: 57.35%; concentration: 3.68 mg L^−1^) [30].

### 2.2. Buffer and Additive Screening Evaluation upon hSCOMTVal108-6His Thermal Stability

A preliminary TSA screening of hSCOMTVal108-6His samples obtained by IMAC was performed in order to select the best signal-to-noise ratio testing different protein concentrations (25–60 µM): 50 µM was established as the ideal protein concentration (Supplementary Materials Figure S1).

The hSCOMT denaturation curves and the respective first derivatives were analyzed to calculate the T_m_ value. A buffer screening of 30 different buffers and salt types (citric acid, sodium acetate, sodium citrate, potassium phosphate, sodium phosphate, MES, sodium cacodylate, PIPES, MOPS, HEPES, ammonium acetate, Tris, imidazole, bicine, CAPS, and glycine-HCl) was tested without and with and NaCl (300 mM and 1 M) and a pH range from 4 to 11.

The T_m_ values determined from each condition were compared with the T_m_ value from the control experiment at which two different buffers were used (Buffer A—10 mM Tris-HCl pH 7.8 and Buffer B—50 mM Tris-HCl pH 7.5, 50 mM NaCl, 2 mM MgCl_2_, and 10 mM DTT). Buffer A was selected due to the stability of the protein during the enzyme activity assays, while Buffer B was selected as it was reported to be successfully applied in crystallization studies of the COMTVal/Met108 soluble isoform [38]. Moreover, both buffers had the addition of 0.5 M of the ionic liquid 1-butyl-3-methylimidazolium chloride ([C4min]Cl) to avoid the precipitation of hSCOMTVal108_6His during its concentration. The incorporation of [C4mim]Cl proved to be highly efficient, ensuring protein stability during the concentration step as demonstrated for other proteins such as periplasmic molybdenum aldehyde oxidoreductase (PaoD) and lysozyme [39,40].

**Figure 4:**
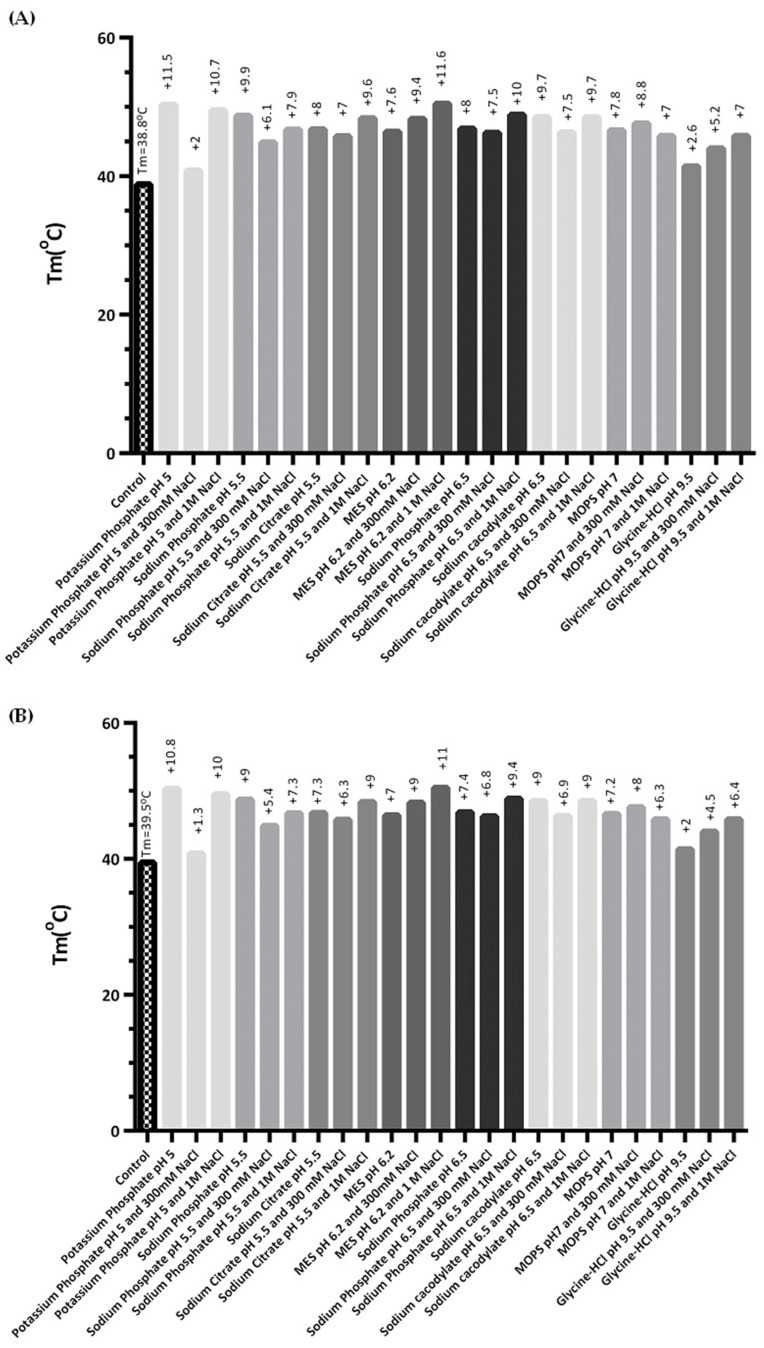
Graphical representation of the Tm values of hSCOMT in the presence of the buffers that gave a clear thermal transition. The control experiment was prepared using (**A**) 10 mM Tris-HCl pH 7.8 and 0.5 M [C_4_min]C**l**. (**B**) 50 mM Tris-HCl pH 7.5, 0.5 M [C_4_mim]Cl, 50 mM NaCl, 2 mM MgCl_2_, and 10 mM DTT.

The melting temperature shift (ΔT_m_) was calculated and the best results are shown in Figure 4. The controls present T_m_ values of 38 and 41 °C: buffer B exhibits a slightly higher value probably due to the presence of NaCl as well as the cofactor MgCl_2_, and the reducing agent DTT was reported to be able to reduce enzyme activity losses [30,41].

Moreover, considering the pH range, buffers presenting a pH below 5 or above 8, with exception of glycine-HCl, presented a poorly defined melting temperature curve, which prevents an accurate T_m_ determination. This finding is corroborated by previous works that showed that the biological activity of hSCOMT is ensured in a pH range between 3 and 8, leading to the exclusion of several buffers (e.g., citric acid, sodium acetate, PIPES, ammonium acetate, HEPES, imidazole, bicine, and CAPS) from further analysis [30,42].

Potassium phosphate, sodium citrate, MES, and sodium phosphate, supplemented by NaCl, present a T_m_ increment between 9 and 11 °C when compared to the controls. In fact, these results are supported by the available literature that describes phosphate-based buffers [32], as well as MES [43,44] and Tris buffers [30], as being the most suitable and often used COMT buffers—the last one was inclusively successfully used in the crystallization of human hSCOMTVal/Met108 [38]. However, although positive ΔT_m_ values were obtained with potassium phosphate, MES, and sodium phosphate, these results were not accurate due to curve interference (Supplementary Materials Figures S2 and S3), and the buffers were not used in further studies. It should be noted that the raw data (non-normalized fluorescence signal vs. temperature) for the best thermofluor assays can be consulted in the spreadsheet of Supplementary Materials 2. Overall, the most suitable stabilizer buffer for hSCOMTVal108-6His was sodium citrate, presenting a positive T_m_ shift of approximately 10 °C, as well as a good melting temperature curve fit (data not shown). Nevertheless, despite being characterized as a powerful protein stabilizer, sodium citrate is also known to be unable to maintain protein biological activity [28,45]. Therefore, we decided to pursue the studies with the addition of trehalose, cysteine, and glycerol (for the stabilization of hSCOMTVal108-6His lysates) using a Tris buffer base (Buffer A: 10 mM Tris-HCl pH 7.8 and 0.5 M [C4mim]Cl).

**Figure 5:**
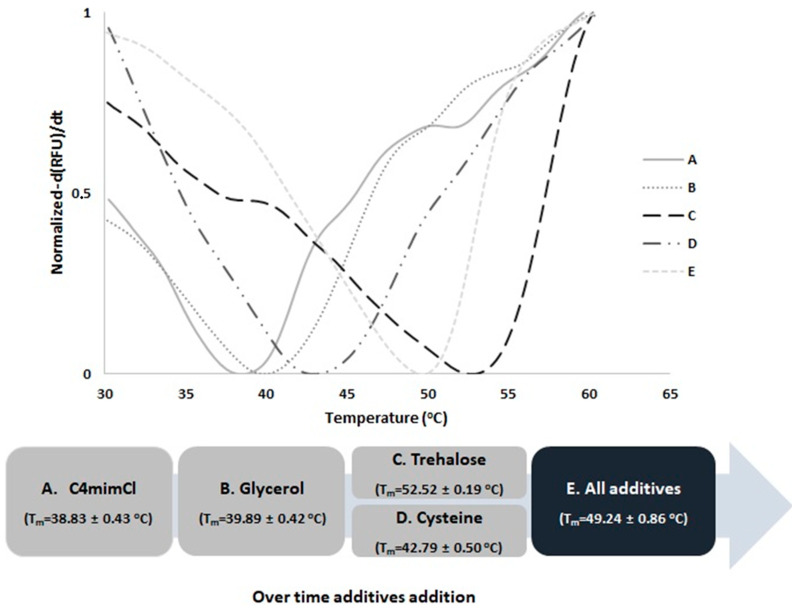
Graphical representation of hSCOMTVal108 normalized first derivate—(dRFU)/dt vs. temperature in the presence of: A. 10 mM Tris-HCl and 0.5 M [C_4_mim]Cl; B. Buffer A and 30% glycerol; C. Buffer B and 150 mM trehalose; D. Buffer B and 100 mM cysteine; E. Buffer A and 150 mM trehalose, 100 mM cysteine and 30% glycerol.

Purified samples of hSCOMTVal108-6His in the referred Buffer A showed an approximate T_m_ value of 39 °C as shown in Figure 5. Once 30% glycerol was added to Buffer A, a shift in the first derivate curve was observed corresponding to a slight T_m_ increment of 1 °C; this corroborates the results reported in Section 2.1 in which the incorporation of 30% glycerol increased the protein activity by approximately 50%.

Similar behavior was detected when 150 mM trehalose and 100 mM cysteine were individually added to buffer A with 30% glycerol with a T_m_ increment of almost 13 °C and 4 °C, respectively. Considering the obtained percentage of activity recovery, both additives were able to maintain protein activity, but cysteine presents a higher increment of almost 100% when compared to trehalose. Therefore, the higher trehalose T_m_ value is probably explained by a synergistic action with glycerol, as previously reported [46].

When all the additives were added together to buffer A, a slight decrease in the best T_m_ value was detected even if a positive 10 °C ∆T_m_ is still observed Therefore, higher hSCOMTVal108-6His stability was achieved using the so-called Buffer E.

### 2.3. Influence of the Storage Temperature and Formulation Validation for hSCOMTVal108-6His Pure Samples

The storage of pure protein samples is a critical step to obtain viable biophysical, enzymatic, and structural insights, as such these techniques might require considerable amounts of time to be performed. Therefore, a stored protein sample should maintain its original biological and functional features for long periods of time, ranging from days to weeks and, in extreme cases, years [47].

The shelf life of a highly thermolabile enzyme such as hSCOMTVal108 is especially related to the intrinsic nature of protein and the storage conditions at an appropriate temperature. In particular, from previous results with the membrane isoform of recombinant human COMT in 10 mM Tris-HCl pH 7.8, 30% glycerol, 100 mM cysteine, and 300 mM trehalose, it was shown that protein remains active for 72 h when stored at −80 °C, presenting also an increment in the protein activity recovery of 125% (data not shown). More recently, the same isoform in the presence of 50 mM Tris-HCl pH 8, 150 mM NaCl, 1 mM MgCl_2_, 7.5 mM of [Ch][DHP], 15 mM cysteine, 5 mM trehalose, and 5% glycerol maintained its activity up to 32.4 h when stored at −80 °C, presenting also an increase in its activity recovery of approximately 140%, with a drastic reduction in the main additive (cysteine and trehalose) concentrations [25]. It was also reported that for a long storage period of 72 h at −80 °C, hMBCOMT still presents an increment of 27% on its original activity recovery. Moreover, if 4 °C was considered to preserve the protein, the addition of low concentrations of [Ch][DHP] maintained the enzyme’s biological activity for 48 h. Therefore, this stabilization formulation can favor later crystallization and bio-interaction trials, while disfavoring the precipitation of the additives present at higher concentrations [48]. Herein, and for the soluble isoform hSCOMTVal108-6His, both 4 and −80 °C temperatures were evaluated after a period of 72 h of storage (Table 1) using protein samples solubilized in both Buffer A and Buffer B with and without glycerol. As shown in Table 1, hSCOMT storage at −80 °C originates smaller T_m_ values in comparison to storage at 4 °C by a factor of 6/7 °C. These data may be indicative of SCOMT undergoing the phenomenon of cold denaturation, which involves protein unfolding caused by cooling the protein from room temperature to lower values [49,50].

However, when glycerol is added, the effect is not so significant, reinforcing its cryo-protective role.

For buffer A, the addition of glycerol favors the stability of the protein at −80 °C compared to storage at 4 °C. However, when a more complex buffer such as B is applied, glycerol increases the T_m_ value of the sample stored at −80 °C, but does not affect the T_m_ value for the sample stored at 4 °C. In fact, the addition of glycerol reveals positive effects in the −80 °C storage, independently, from the used buffer registering a Tm increasing in range from 3 to 4 °C. This behavior has already been observed for proteins such as β-lactoglobulin, lysozyme, and amylase, among others [11,51,52].

On the other hand, at 4 °C, the addition of 30% glycerol seems to destabilize the native folding of the protein favoring its denaturation. This effect is more pronounced in the presence of 10 mM Tris-HCl buffer with 0.5 M [C_4_mim][Cl].

**Figure 6:**
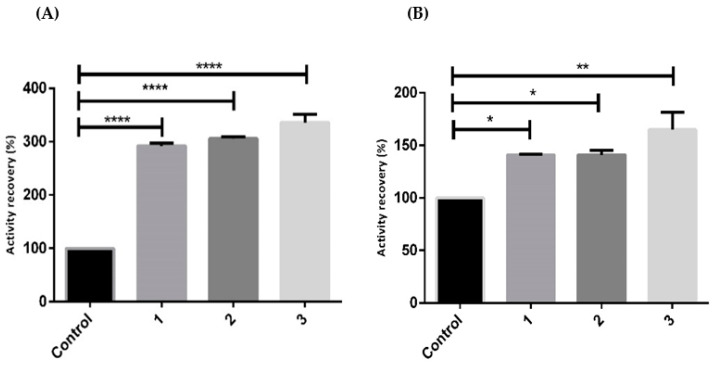
Percentage of hSCOMT activity recovery in the absence (control) and presence of: 1. 15 mM cysteine, 5 mM trehalose, and 5% glycerol; 2. 1 and 10 mM [Ch][DHP]; 3. 1 and 10 mM [C_4_mim]Cl during a 12 h period at (**A**) 4 °C and (**B**) −80 °C. Each value represents the mean of 3 independent samples (*p*-value * < 0.05; ** < 0.01; **** < 0.0001).

Once, during hSCOMTVal108-6His crystallization trials, the formation of cysteine crystals was observed when a concentration of 100 mM was used, lower concentrations of additives (15 mM cysteine, 5 mM trehalose, and 5% glycerol) were applied in order to stabilize the enzyme as successfully tried with the membrane isoform hMBCOMTVal158 [25]. Moreover, two distinct Ionic Liquids (ILs) classes, choline, with [Ch][DHP], and imidazole, with [C_4_mim]Cl, were evaluated in hSCOMTVal108-6His lysates in terms of percentage of activity recovery at the two storage temperatures (4 °C and −80 °C), as shown in Figure 6. The addition of the mentioned additives, even at a low concentration, was able to maintain and even increase the percentage of hSCOMTVal108-6His activity recovery, with this increase being more significant at 4 °C. As discussed above, and in good agreement with Tm data depicted in Table 1, this behavior seems to be indicative of the cold denaturation phenomenon, where the lowest temperature in the study leads to a decrease in hSCOMT biological activity [49,50].

Similar behavior was observed when ILs were introduced to the storage protein buffer, with [C_4_mim]Cl presenting slightly better results than [Ch][DHP], in opposition to those observed for the membrane isoform (Figure 6) [14]. Concerning *p*-values, the results were highly significant for the protein when stored at 4 °C for all the tested conditions (*p*-value < 0.0001), and significant when the protein was stored at −80 °C (*p*-value < 0.05 and *p*-value < 0.01).

**Figure 7:**
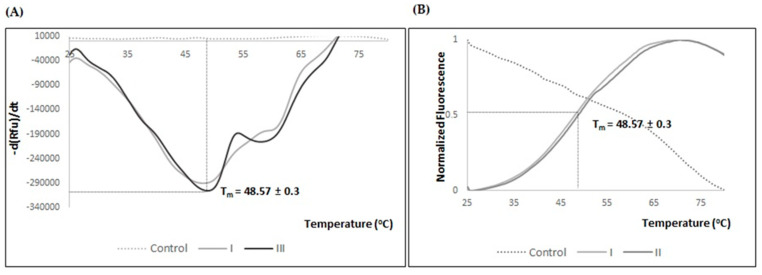
(**A**) TSA melting curve of hSCOMTVal108-6His from thermal stability fluorescence data of first derivate (d(Rfu)/dt) curves in 150 mM NaCl, 50 mM Tris, 1 mM MgCl_2_, 15 mM cysteine, 5 mM trehalose, 5% glycerol, and 10 mM [C_4_mim]Cl. (**B**) Normalized TSA melting curve of hSCOMTVal108-6His from thermal stability fluorescence data of first derivate (d(Rfu)/dt) curves. Control sample as 10 mM Tris-HCl pH 7.8 and 0.5 M [C_4_mim]Cl.

To confirm the increased stability of the protein in the buffer 150 mM NaCl, 50 mM Tris pH 7.8, 1 mM MgCl_2_, 15 mM cysteine, 5 mM trehalose, 5% glycerol, and 10 mM of [C_4_mim]Cl, a TSA experiment was performed with purified fractions of hSCOMTVal108-6His (Figure 7). The obtained results presented a T_m_ value of approximately 49 °C similar to the one obtained when the protein was stored with the same additives at higher concentrations. This observation shows that lowering the additive concentrations does not affect the observed ΔT_m_ of 10 °C between the initial storage buffer A (10 mM Tris-HCl pH 7.8 and 0.5 M [C_4_mim]Cl and the optimized buffer.

**Figure 8:**
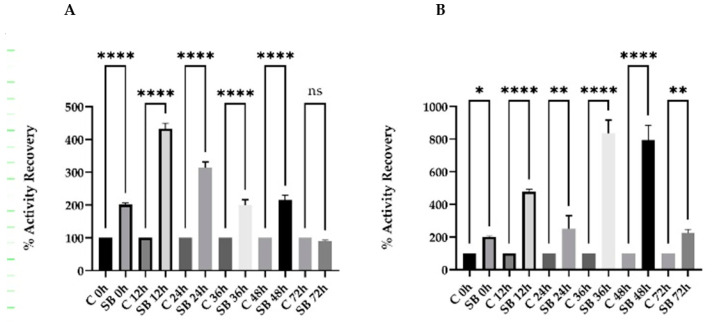
Percentage of recovery of hSCOMT activity in the presence and absence of Stabilizer Buffer (**A**) 4 °C; (**B**) −80 °C for a period of 0, 12, 24, 36, 48, and 72 h. Each value represents the average of 3 independent samples. The Control Buffer (**C**) and Stabilizer Buffer (SB) were composed, respectively, by 50 mM of Tris-Cl pH 7.8, 50 mM NaCl, 1 mM MgCl_2_, 5 mM imidazole, and 50 mM of Tris-Cl pH 7.8, 150 mM NaCl, 1 mM MgCl_2_, 15 mM cysteine, 5 mM trehalose, 5% glycerol, and 10 mM [C_4_min]Cl. (*p*-value * < 0.05; ** < 0.01; **** < 0.0001).

Subsequently, we analyzed the recovery of the hSCOMT enzymatic activity in pure samples and concentrated on the control and “ideal” buffer (Figure 8). The data obtained are statistically significant and indicate that we were able to recover the enzyme activity at a higher percentage when stored at −80 °C between 36 and 48 h when compared to 4 °C for 12 and 24 h. Moreover, the use of the stabilization buffer (SB) in the recombinant protein concentration step subsequent to IMAC stores the target sample at 4 °C for 24 h to reach a maximum yield in terms of bioactivity of 823% (control—176%) with a degree of purification of 15 (about five times higher when compared to the control: 3.3. Additionally, −80 °C storage with the stabilization buffer extends the storage window to 48 h, reaching a maximum yield of 1122% (control—114%) and a degree of purification of around 21.5 (control—2.7). These results represent a huge increase in the activity recovery and purification factor when compared to what is described in the literature. As reported, the low stability and high activity of cold-adapted enzymes at low temperatures imply a flexible enzyme structure, such as hSCOMTVal108. Considering that the catalytic residues are conserved between cold-adapted and thermostable protein homologs, the referred flexibility should be caused by some other distinct residue regions [49,50].

To understand if the new formulation can promote a reversible denaturation of SCOMT, we assessed the recovery of hSCOMT activity in the presence and absence of the stabilizing buffer after heating at 70 °C, followed by cooling to 4 °C. In the presence of the stabilizing solution, heating the enzyme to 70 °C causes a highly significant loss of activity (Supplementary Materials Figure S4). Cooling the SCOMT sample to 4 °C does not recover the enzyme activity. Thus, even in the presence of the stabilization solution, the denaturation of the enzyme is completely irreversible as described earlier [38]. These outputs can undoubtedly compromise the assessment of thermodynamic parameters such as delta G.

**Figure 9:**
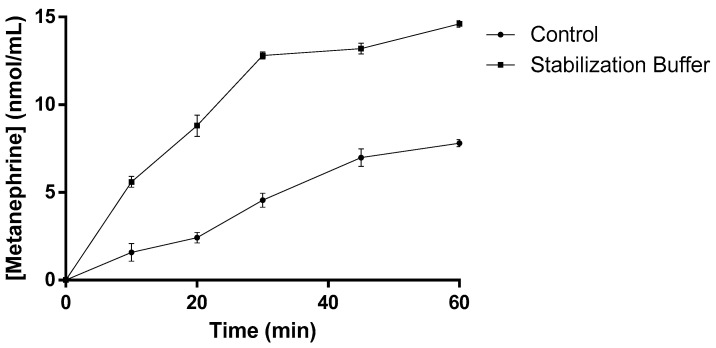
Metanephrine concentration over time obtained in the absence and presence of the stabilization solution formulated in the course of this work.

Finally, the productivity of the SCOMT enzyme was analyzed for up to 60 min in the control formulation and in the optimized buffer with stabilizers. The enzymatic reaction under analysis involved the methylation of the substrate epinephrine into metanephrine, in the presence of its major cofactors Mg^+2^ and S-adenosyl-L-methionine (Figure 9).

Productivity analysis confirmed that thermolabile COMT presents an ~2-fold higher efficacy of *O*-methylation in the presence of the stabilizing solution relative to in its absence (Figure 9). Overall, the higher productivity of an extremely thermolabile COMT can be achieved by the stabilization of the enzyme in the presence of additives explored in this study. This suggests that the enzyme can be utilized in the presence of the additives for various biotechnological applications.

## 3. Materials and Methods

### 3.1. Chemicals

Ultrapure reagent-grade water for preparative and analytical chromatographic systems was obtained with a Milli-Q system from Merck (Darmstadt, Germany). The components of the mobile phase applied in the HPLC system with coulometric detection, Agilent Technologies (Santa Clara, CA, USA) such as citric acid monohydrate, sodium octyl sulfate (OSA), and the reagents used to assess the hSCOMTVal108 specific activity, such as S-adenosyl-L-methionine (SAM), epinephrine (bitartrate salt), and DL-metanephrine hydrochloride, were purchased from Merck KGaA (Darmstadt, Germany). The organic solvents such as methanol and acetonitrile were obtained from VWR (Radnor, PA, USA). The additives used in the stabilization of the hSCOMT enzyme such as cysteine (L-), trehalose, and 1-butyl-3-methylimidazolium chloride ([C_4_mim]Cl) were also purchased from Merck KGaA, and glycerol was obtained from VWR (Radnor, PA, USA). Both Pierce BCA Protein Assay Kit and Protein Thermal Shift™ Dye Kit were obtained from Thermo Fisher Scientific (Waltham, MA, USA). Regarding the components used in the biosynthesis step, either on a small or large scale: agar was obtained from Pronadisa (Basel, Basel-Stadt, Switzerland); yeast nitrogen base and yeast extract were obtained from Himedia (Thane West, MH, India); and peptone was obtained from Becton, Dickinson and Company (Franklin Lakes, NJ, USA). Common salts such as dipotassium phosphate (K2HPO4) and sodium acetate anhydrous (NaH_2_PO_4_) were obtained from Panreac (Barcelona, Catalonia, Spain), and additionally, monopotassium phosphate (KH_2_PO_4_) and magnesium chloride (MgCl_2_) were obtained from Chem-Lab (Zedelgem, West Flanders, Belgium). All chemicals used were of analytical grade and were applied directly without further purification.

### 3.2. Recombinant hSBCOMT Biosynthesis and Recuperation

The biosynthesis of human recombinant SCOMTVal108-6His protein was performed according to technical details described by Pedro and coworkers [30]. Succinctly, recombinant *Komagataella pastoris* X33 was grown for 72 h at 30 °C in yeast extract peptone dextrose (YPD) medium plates with 200 μg/mL of zeocin. A single colony was inoculated in 100 mL of buffered minimal glycerol medium (BMGH), and cells were grown at 30 °C and 250 rpm to a cell density of 6.0 units. Subsequently, an aliquot was transferred into 100 mL of buffered minimal methanol medium (BMMH) in flasks with a total capacity of 500 mL. The optical capacity at 600 nm was fixed to 1.0 unit and the operation conditions were fixed at 30 °C and 250 rpm. After 24 h of growth, cells were collected by centrifugation (1500× *g*, 10 min, 4 °C). The cells were resuspended in lysis buffer (50 mM Tris pH 8, 150 mM NaCl, 10 mM DTT, and 1 mM MgCl2) supplemented with protease inhibitor cocktail. To disrupt the cells, a mechanical treatment with glass beads was applied [30]. After centrifugation (500× *g*, 5 min, 4 °C), the pellet obtained was resuspended in the lysis buffer, but without DTT.

### 3.3. Purification of hSCOMTVal108-6His by Immobilized Metal Affinity Chromatography

The purification of the human recombinant SCOMTVal108-6His was performed according to the procedure described by Pedro et al. [30]. Briefly, chromatographic experiments were performed in Äkta Start system with UNICORN 6.1 software (GE Healthcare, Sweden). The chromatographic runs were performed at 20 °C on a HisTrap^TM^ FF crude (5 mL) (GE Healthcare, Sweden). The column was equilibrated under the same conditions described by our research group in [30]. The resuspended pellet in the equilibrium buffer was applied onto the column using a sample pump at a flow rate of 0.5 mL min^−1^. Under the conditions described and after the elution of proteins that do not have any interaction with the column, the concentration of imidazole was increased in a step mode of 50, 70, 300, and 500 mM. All the elution steps were performed with 5 CVs at 1 mL min^−1^. Purified hSCOMTVal108-6His were pulled at 300 mM of imidazole, concentrated, and desalted with Vivaspin concentrators (10,000 MWCO) (Sartorius, Gottingen, Germany). The purified samples were stored at 4 °C until use for further assays.

### 3.4. hSCOMT Stabilization Studies

The stabilizers understudy was added separately with a range of concentrations as shown in Table 2. Typically, the concentration of total protein in the lysates containing the target enzyme was normalized to the value of 1 mg ml^−1^. The initial experiences were performed primarily at 4 °C with the target stabilizer concentrations for 12 h. Enzymatic hSCOMT activity levels were evaluated by the capacity of the hSCOMTVal108-6His to convert epinephrine to metanephrine, as described previously [32]. For each concentration of stabilizer under evaluation, the percentage of the enzyme activity recovery after 12 h of storage at 4 °C was quantified by the ratio between the specific activity with and without stabilizers. Additionally, and using the same methodology described above, we carried out several studies analyzing the storage time of 12, 24, 48, and 72 h at temperatures of 4 °C and −80 °C. All data analysis was performed using Prism 6 (GraphPad Software Inc. San Diego, CA, USA).

The productivity of the SCOMT enzyme was analyzed for up to 60 minutes in the absence (control) and presence of formulation (optimized buffer with stabilizers). The enzymatic reaction involved the methylation of the substrate epinephrine into metanephrine, in the presence of its major cofactors Mg^+2^ and S-adenosyl-L-methionine. For each time, three independent replicates were performed. Control Buffer: 150 mM NaCl, 50 mM Tris, and 1 mM MgCl2; and Stabilizer Buffer: 150 mM NaCl, 50 mM Tris, 1 mM MgCl2, 15 mM Cysteine, 5 mM Trehalose, 5% Glycerol, and 10 mM [C4min]Cl [53].

### 3.5. Thermal Shift Assay (TSA)

The effect on protein stability of the two initial buffers—Buffer A (10 mM Tris-HCl pH 7.8, and 0.5 M [C4mim]Cl) and Buffer B (50 mM Tris-HCl pH 7.5, 50 mM of NaCl, 2 mM MgCl_2_, 10 mM DTT, and 0.5 M [C4mim]Cl)—was tested in MicroAmp^®^Fast 96-well reaction plates (Thermo Fisher Scientific, Waltham, MA, USA): a sample of 20 µL was used with 17 µL of 50 µM SCOMT and 3 µL of Protein Thermal Shift™ Dye Kit (Thermo Fisher Scientific, Waltham, MA, USA).

A second TSA experiment was designed to evaluate the different additives in the stabilization of hSCOMTVal108-6His. The experiment was conducted similarly to the previous one using MicroAmp^®^Fast 96-well reaction plates (ThermoFisher Scientific, Waltham, MA, USA) and a sample of 20 µL with 10 µL of the desired buffer, 5 µL of the protein purification buffer (10 mM Tris-HCl, pH 7.8), 2 µL of 50 µM hSCOMTVal108-6His (protein concentration in the plate well: 5 μM), and 3 µL of Protein Thermal Shift™ Dye Kit (Thermo Fisher Scientific, Waltham, MA, USA).

Both TSA experiments were executed using 2 min cycles of 1% increments between 25 °C and 95 °C in a StepOnePlusTM Real-Time PCR System (ThermoFisher Scientific, Waltham, MA, USA). Data processing and data analysis were performed in Protein Thermal Shift^TM^ Software (Thermo Fisher Scientific, Waltham, MA, USA). T_m_ values were calculated by the first derivative of the raw fluorescence data.

## 4. Conclusions

Due to its important biological function and potential therapeutical action, COMT remains today a central focus of several studies. The rapid loss of its biological activity, probably due to the loss of its native conformation, is a setback that needs to be overcome. Thus, the present study focuses on profiling the thermal stability of hSCOMTVal108-6His, determining putative additives to supplement the storage buffer in order to enhance protein stability. From the performed assays, it was shown that cysteine, trehalose, and glycerol were able to maintain and even increase the percentage of hSCOMTVal108-6His lysate activity recovery to 125% during a period of 72 h when the protein was stored at −80 °C. Moreover, using these additives combined with the ionic liquid [C_4_mim]Cl, even at low concentrations (15 mM cysteine, 5 mM trehalose, 5% glycerol, and 10 mM [C_4_mim]Cl), the percentages of activity recovery increase for both storage temperatures: 200% (4 °C) and 70% (−80 °C) for a period of 12 h. Interestingly, the new hSCOMTVal108 stabilization buffer applied during the protein concentration step allows a maximum bioactivity yield of 823% and 1122% at 4 °C for 24 h and −80 °C for 48 h to be reached, respectively. These results were further confirmed by TSA in which an increment of 10 °C in the T_m_ was observed for the hSCOMTVal-6His lysates and validated in purified fractions, supporting the improvement in protein stability. These promising results prompt additional biophysical and structural studies in order to better characterize the protein COMT as a viable anti-Parkinson’s disease target.

## Figures and Tables

**Table 1:** Influence of glycerol on Tm values of hSCOMT when in storage at 4 °C and −80 °C.

Buffer	Additive (%(*v*/*v*))	Storage Temperature (°C)	T_m_ (°C)
A	- -	4 °C	44.2 ± 1.0
		−80 °C	37.6 ± 0.2
	30% glycerol 30% glycerol	4 °C	40.6 ± 0.2
		−80 °C	40.4 ± 0.4
B	- -	4 °C	43.1 ± 0.8
		−80 °C	36.8 ± 0.5
	30% glycerol 30% glycerol	4 °C	44.1 ± 0.6
		−80 °C	41.6 ± 0.3

Note: Buffer A. 10 mM Tris-HCl pH 7.8 and 0.5 M [C_4_mim]Cl; Buffer B. 50 mM Tris-HCl pH 7.5, 50 mM NaCl, 2 mM MgCl_2_, 10 mM DTT, and 0.5 M [C_4_mim]Cl.

**Table 2:** hSCOMT stabilizers features and concentrations.

Stabilizer	Feature	Concentration Range
Cysteine	Stabilization of disulfide bonds	75 to 500 mM
Trehalose	Thermal stabilizers/promote protein folding and refolding	10 to 350 mM
Glycerol	Cryo-protector	5 to 50%
[C4mim]Cl	Thermal stabilizer/aggregation behavior	10 mM *

* Note: [C4mim]Cl only had a concentration tested due to previous optimizations [25].

## Data Availability

Data is contained in the article.

## Supplementary Materials

The following supporting information can be downloaded at: https://www.mdpi.com/article/10.3390/ijms232012298/s1.
