# Supplementary material for: Thermofluor-Based Optimization Strategy for the Stabilization of Recombinant Human Soluble Catechol-O-Methyltransferase
Source: Int J Mol Sci. 2022 Oct 14;23(20):12298. doi: 10.3390/ijms232012298 (PMC9603843; doi:10.3390/ijms232012298)
Supplement: Supplementary file 1 [file ijms-23-12298-s001.zip › Suplementary Material 1.pdf]

International Journal of
Molecular Sciences

MDPI

Supplementary material

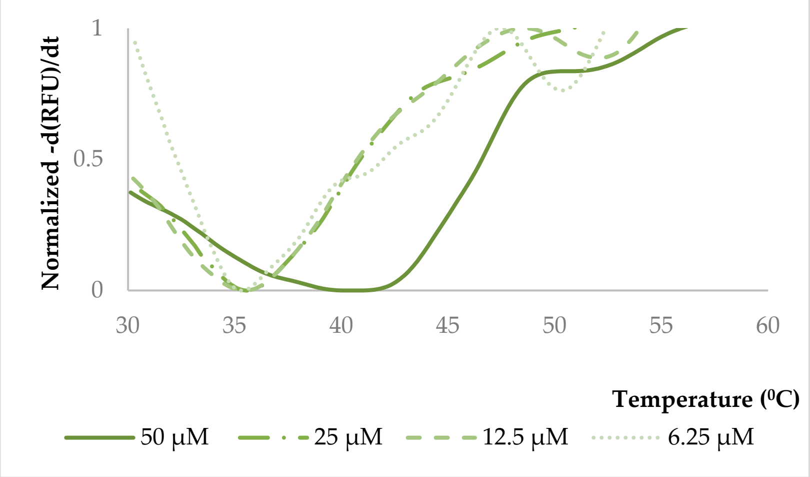

Supplementary Figure S1. Normalized TSA melting curve of hSCOMTVal108-6His from thermal stability fluorescence data of first derivate (d(Rfu)/dt) curve in 50 mM of Tris-HCl pH 7.5, 50 mM of NaCl, 2 mM of $MgCl_2$, 10 mM of DTT and 0.5M [C4mim]Cl at different at different stock hSCOMT concentrations (50; 25; 12.5 and 6.25 µM).

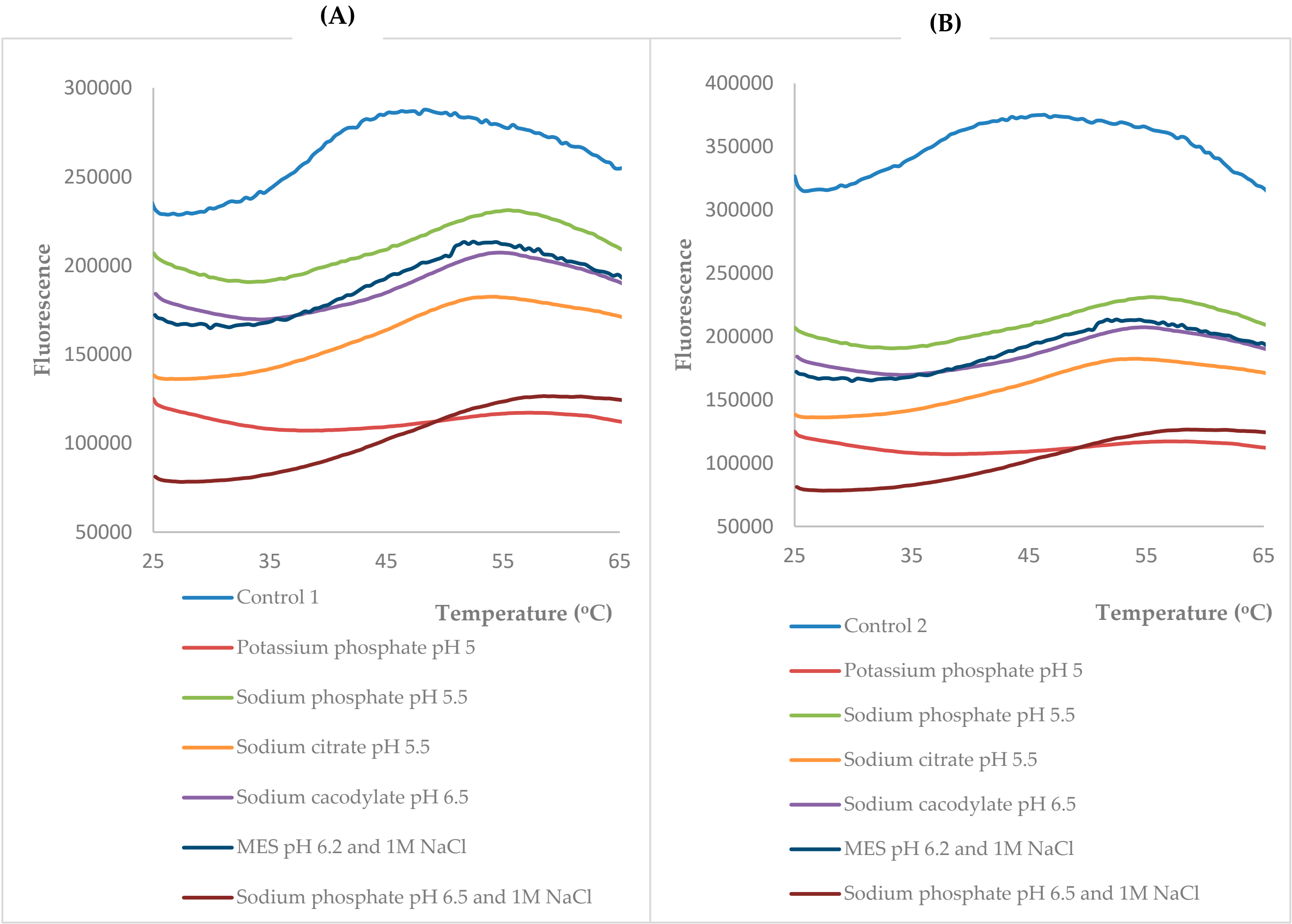

Supplementary Figure S2. TSA melting curve of hSCOMTVal108-6His from thermal stability fluorescence data vs temperature buffer screen in **(A)** Control 1- 10 mM of Tri-HCl and 0.5 M [C4mim]Cl; **(B)** Control 2- 50 mM of Tris-HCl pH 7.5, 50 mM of NaCl, 2 mM of $MgCl_2$, 10 mM of DTT and 0.5M [C4mim]Cl.

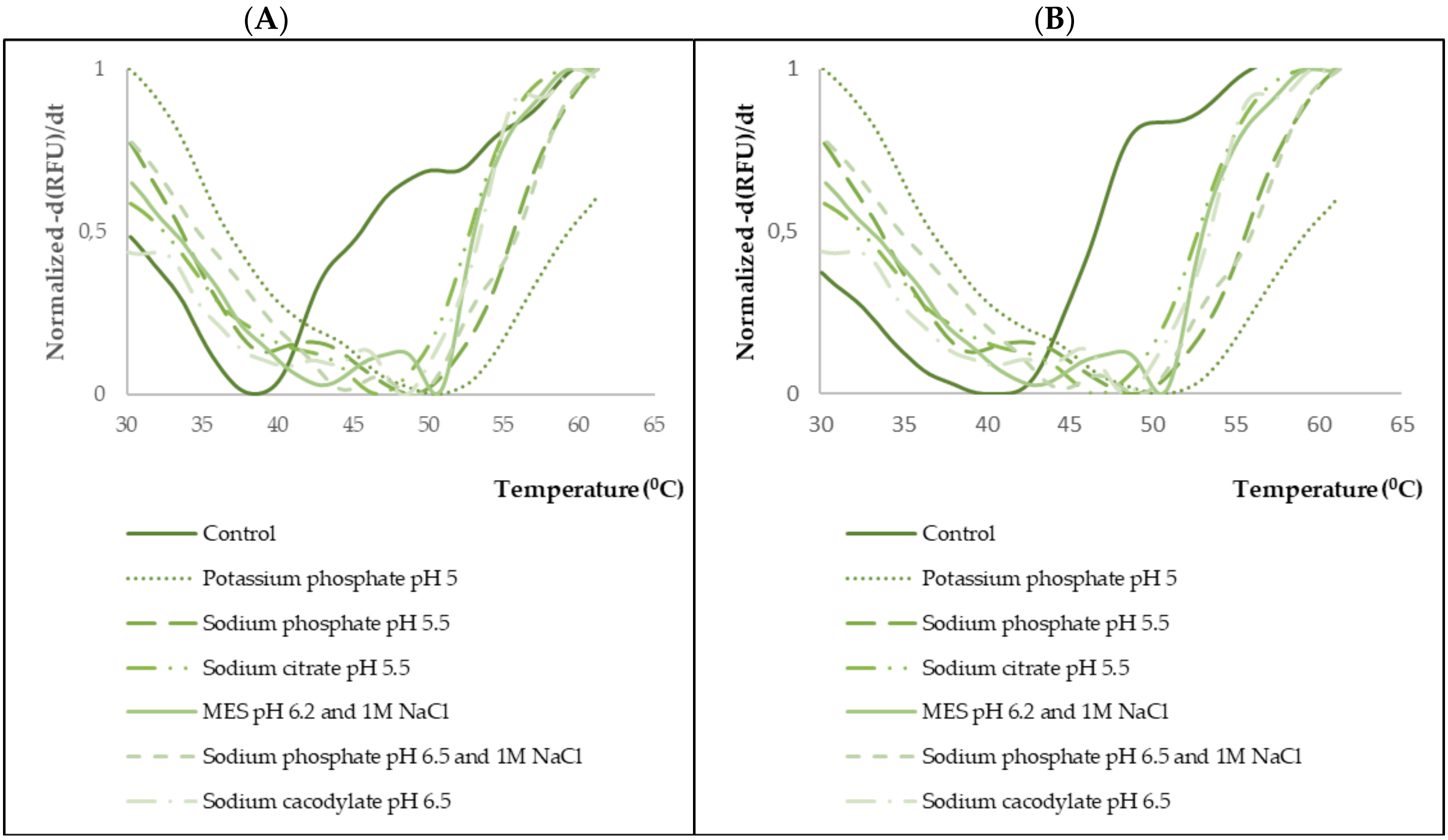

Supplementary Figure S3. (A) Normalized TSA melting curve of hSCOMTVal108-6His from thermal stability fluorescence data of first derivate (d(Rfu)/dt) curve buffer screen in 10 mM Tris-HCL pH 7.8 and 0.5 M [C4mim]Cl; **(B)** with 50 mM of Tris-HCl pH 7.5, 0.5 M [C4mim]Cl, 50 mM of NaCl, 2 mM of $MgCl_2$ and 10 mM of DTT.

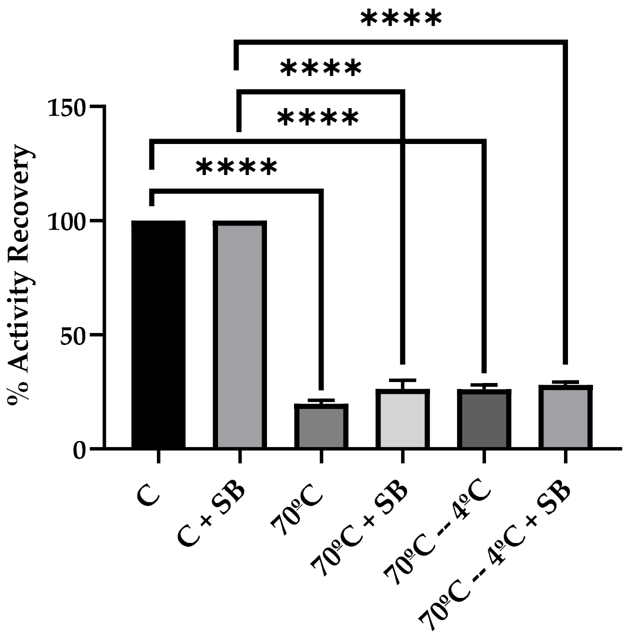

Supplementary Figure S4. Percentage of recovery of hSCOMT activity in the presence and absence of Stabilizer Buffer after heating at 70°C and heating at 70°C and progressive cooling to 4°C. Each value represents the average of three independent samples (****$p < 0.0001$ when compared with the control formulation). Control Buffer (C): 150 mM NaCl, 50 mM Tris, 1 mM $MgCl_2$ and Stabilizer Buffer (SB): 150 mM NaCl, 50 mM Tris, 1 mM $MgCl_2$, 15 mM Cysteine, 5 mM Trehalose, 5% Glycerol, 10 mM $[C_4minCl]$.
